# Changes in plasma biochemical parameters and hormones during transition period in Beetal goats carrying single and twin fetus

**DOI:** 10.14202/vetworld.2020.1025-1029

**Published:** 2020-06-05

**Authors:** Jyotsana Madan, Sonia Sindhu, Manoj Kumar Rose

**Affiliations:** Department of Veterinary Physiology and Biochemistry, Lala Lajpat Rai University of Veterinary and Animal Sciences, Hisar, Haryana, India

**Keywords:** biochemical, enzymes, goats, hormones, transitional period

## Abstract

**Aim:**

The study was undertaken to find out the changes in biochemical and hormonal profile during transition period in single- and twin-bearing Beetal goats.

**Materials and Methods:**

Beetal goats are reared in North India for milk and meat purposes, popularly called poor man’s cow, serving as a source of livelihood. The present study was conducted on Beetal goats, maintained at a goat farm, Lala Lajpat Rai University of Veterinary and Animal Sciences, Hisar, for characterization of plasma biochemical and hormonal changes during transitional phase in pregnant goats carrying single and twin fetus. The animals selected were expected to have parturition at the same time, to avoid environmental variation effects, and divided into two groups: Group 1 (carrying single fetus, n=14) and Group 2 (twin fetus, n=6). Blood samples were collected from goats prepartum (−30 days and −15 days), 0 day, and postpartum +15 days and +30 days, and plasma was separated for the analysis of biochemical metabolites and hormones by commercially available kits.

**Results:**

Analysis of data revealed a significant decrease in triglyceride and cholesterol concentration during postpartum days. A significant increase in alanine aminotransferase (ALT) concentration was observed at the day of parturition. Furthermore, there was a significant (p≤0.05) decrease in ALT and aspartate aminotransferase concentration in goats at the 15^th^ day and 30^th^ day after parturition in both groups. A significant (p≤0.05) lower concentration of total protein and globulins was observed during late pregnancy and on the day of parturition, with a subsequent elevation during postpartum period (15 days and 30 days). Progesterone concentration was highest at −30 days prepartum and decreased sharply at parturition and thereafter postpartum, both in single- and twin-bearing goats. Progesterone level was lower in twin-bearing goats as compared to single-bearing goats at 0 day and postpartum days. No significant changes were observed in sodium, potassium, and chloride concentration during transitional period in goats.

**Conclusion:**

Transition period blood metabolite profile changes due to physiological need of mother and fetus were more prominent in metabolites regulating energy requirements and in goats bearing twin fetus.

## Introduction

Under subsistence agriculture, goats are known to be favorable ruminant animals compared to other ruminants because of their unique ability to adapt and adjust to unfavorable environmental conditions. The reproductive performance of goats is the most valuable trait that determines sustainability and continual use as a valuable resource to improve livelihood [1]. The reproductive status varies with different stressors including transition phase of animals. The growing fetus’ demands and lactogenesis associated with mammary gland activity in postpartum period lead to enhanced requirement of nutrient during the transitional phases. Such changes require dramatic physiological and biochemical adaptations during the above said period [2]. Therefore, transition period is the most crucial period in the female productive life, having significant effects on fertility, health, milk yield, and profitability, more particularly in high-yielding animals. It has been already reported that changes in various physiological conditions such as age, breed, metabolic disorders, and reproductive status and managemental conditions such as season, stress caused by management, road transportation, or temperature have direct implications on animal hematobiochemical profile [3]. These changes also affect the feed intake and animal’s endocrine status, which when altered can have a detrimental effect on lactogenesis.

These physiological changes can also occur in the female during the transitional period and may serve as the basis for differentiation between normal physiological and pathological metabolic changes, through analysis of metabolic profiles. Blood components such as glucose, proteins, urea, and non-esterified fatty acid can be considered as a marker to assess energy, health, and nutritional status of animals. Energy and lipid mobilization studies and oxidative stress have been specified in goats; however, there are limited numbers of routine reports on biochemical attributes during metabolism studies in goats. Furthermore, reports are very scanty to Indian breeds and difference in physiology with respect to twins and single kid bearing goats, particularly in Beetal breed. Blood profile and production performance can vary with twin or single fetus in goats [4]. Therefore, physiological blood serum-specific chemical reference parameters and their variation should be established in purebred and crossbred dairy female goats under intensive farming system [5,6].

This knowledge can be used for the purposes of diagnosis and prognosis of diseases, for criteria of adaptability, as well as to elucidate many other physiological mechanisms in goats [7]. Hence, the present study was conducted to assess some blood biochemical parameters, electrolytes, enzymes, and hormone level of Beetal goats with single and twin fetus during transition period.

## Materials and Methods

### Ethical approval

The study was conducted following approved guidelines of the Institutional Animal Ethics Committee, Lala Lajpat Rai University of Veterinary and Animal Sciences (LUVAS), Hisar.

### Location of study and experimental animals

The present study was conducted in the Department of Veterinary Physiology And Biochemistry from September, 2015 to November, 2015. The experimental animals were maintained at the goat section, Animal Genetics and Breeding, LUVAS, Hisar, and kept under same managemental practices. The effects of fetal number (single or twin) on blood metabolites and hormone levels during prepartum and post-parturition were studied on 20 Beetal goats in their first to third lactation. The samples were taken during the months of September and October, with the dry temperature ranging from 21.2 to 27.8°C, while the wet temperature was between 19.6 and 20.4°C. Wind velocity was ranging between 2.7 and 10.8 km/h. The animals were divided into two groups, namely, Group 1 (carrying single fetus, n=14) and Group 2 (twin fetus, n=6). Around parturition, the blood samples were taken on days −30 and −15 (before kidding) and on day 0 and +15 and +30 days post-kidding. Approximately 5 ml of whole blood was collected from jugular vein in ethylenediaminetetraacetic acid. Plasma was separated by centrifugation at 3000 rpm for 15 min and stored at −20°C in deep freeze until analyzed for biochemical parameters.

### Biochemical and hormonal analysis

The biochemical metabolites (total protein, albumin, glucose, cholesterol, triglycerides, aspartate aminotransferase (AST), alanine aminotransferase (ALT), chloride, and urea) were analyzed using Erba biochemical kit (ERBA Diagnostics, GmbH, Germany) on semi-automated clinical chemistry analyzer (AGAPPE Diagnostics, India). Electrolytes (sodium and potassium) were measured by flame photometry. Hormones (estrogen and progesterone) were measured by ELISA kits supplied by XEMA (Russia).

### Statistical analysis

The data obtained were analyzed statistically with the help of SPSS version 23 (IBM Corp., NY, USA) software using one-way ANOVA followed by Duncan’s multiple range tests. The data are expressed as mean ± SD with significance level p≤0.05.

## Results and Discussion

The results of blood biochemical parameters are presented in Table-1. Most of the biochemical parameters (total protein, albumin, globulin, cholesterol, sodium, potassium, ALT, and AST) did not vary significantly (p≤0.05) between the groups during the whole period of study. The glucose concentration was significantly (p≤0.05) higher in Group 1 than in Group 2 at −30 days, indicating a higher demand of energy in twin-bearing goats during prepartum period. The higher level of plasma glucose was maintained in Group 1, though statistically non-significant, during the whole period of study. Further study within the next days revealed a significant increase of glucose level at day 0 in both groups as compared to prepartum period and the value was maximum at 30 days postpartum in single producing goats, whereas it remained stable at 15 and 30 days postpartum in twin producing goats.

**Table-1:** Plasma biochemical parameters in single- and twin-bearing Beetal goats during transition period.

Parameters	Group	−30 days	−15 days	Day 0	Day 15	Day 30
Glucose (mg/dl)	Single	47.03±4.46^ax^	46.47±1.69^a^	58.31±3.51^b^	57.44±2.45^b^	62.38±3.51^b^
	Twin	35.02±0.91^ay^	40.32±3.56^b^	57.5±4.23^b^	62.66±5.27^b^	57.84±2.48^b^
Cholesterol (mg/dl)	Single	50.07±3.15^a^	38.98±3.08^b^	44.18±2.75^ab^	39.82±1.88	36.97±2.45^b^
	Twin	47.91±3.36^a^	35.47±2.17^b^	43.78±4.26^a^	36.15±5.04^ab^	37.24±3.09^ab^
Triglyceride (mg/dl)	Single	21.74±1.26^a^	19.86±1.79^ax^	14.68±1.18^b^	15.81±1.37^b^	10.66±0.75^c^
	Twin	26.12±5.35^a^	34.5±5.8^ay^	15.58±3.36^b^	13.08±3.28^b^	9.93±0.68^b^
Total protein (g/dl)	Single	6.44±0.19^a^	7.01±0.19^b^	5.93±0.34^a^	7.47±0.28^b^	7.75±0.28^b^
	Twin	6.64±0.38^b^	6.14±0.31^b^	5.34±0.33^a^	6.79±0.45^b^	7.06±0.22^b^
Albumin (g/dl)	Single	2.53±0.11^b^	2.51±0.08^b^	2.83±0.11^a^	2.16±0.08^c^	2.01±0.13^c^
	Twin	2.31± 0.17^a^	2.44±0.09^a^	2.95±0.12^b^	2.07±0.12^a^	1.99±0.17^a^
Globulin (g/dl)	Single	3.91±0.21^b^	4.49±0.21^b^	3.04±0.31^a^	5.34 ±0.31^b^	5.8±0.29^b^
	Twin	4.51±0.3^b^	3.7±0.32^b^	2.38±0.42^a^	4.72±0.52^b^	5.06±0.19^b^
Urea (mg/dl)	Single	25.94±2.75^ax^	43.99±2.31^b^	39.05±2.4^b^	32.38±2.07^c^	34.72±2.29^c^
	Twin	34.79±3.22^ay^	50.88±2.48^b^	42.3±2.91^b^	31.34±3.46^a^	31.21±1.16^a^

Values within rows^(a,b,c)^ and columns^(x,y)^ with different superscripts are statistically different (p<0.05)

The level of triglycerides was significantly higher (p≤0.05) on day −15 in twin producing goats than singlet bearing goats while at days −30 and 0, it was non-significantly higher in Group 2. Thereafter, a reversion trend was observed between the groups postpartum, although it was statistically non-significant. A significant decrease in cholesterol and triglyceride concentration was observed at day 0 and subsequently at 15 and 30 days postpartum in both groups when data were compared between days, within the groups.

A significant (p≤0.05) lower concentration of total protein and globulins was observed on the day of parturition, and then, a subsequent elevation was observed during postpartum period (15 days and 30 days) in both groups.

The urea concentration varied non-significantly between groups across the period of study, except at day −30 where twin-bearing goats have significantly higher level of urea than single-bearing animals. Further, the study within the group revealed that plasma urea increased significantly at 15 days prepartum but decreased non-significantly around parturition and then declined significantly at 15 days postpartum and remained stable till 30 days postpartum. The results revealed no significant variation in sodium and chloride concentration in both groups (Table-2). Although there was a decrease in potassium concentration in plasma at 0 day and peripartum 15 and 30 days, the values were in normal range in both groups.

**Table-2:** Plasma electrolytes in single- and twin-bearing Beetal goats during transition period.

Parameters	Group	−30 days	−15 days	Day 0	Day 15	Day 30
Sodium (mEq/l)	Single	158.97±1.45^a^	156.88±0.95^a^	155.81±2.14^a^	153.06±3.98^ab^	148.69±3.89^b^
	Twin	158.3±3.47^a^	156.11±1.81^a^	163.65±2.24^a^	143.08±6.35^b^	156.7±9.79^a^
Potassium (mEq/L)	Single	4.95±0.11^a^	4.67±0.09^a^	4.25±0.08^b^	3.79±0.08^b^	4.08±0.1^b^
	Twin	4.84±0.25^a^	4.59±0.22^a^	4.22±0.78^ab^	3.94±0.23^b^	4.12±0.11^b^
Chloride (mEq/L)	Single	107.59±1.80^a^	102.87±7.07^a^	109.6±1.49^a^	105.43±2.13^a^	103.6±2.85^a^
	Twin	105.9±3.93^a^	110.4±1.3^a^	110.85±1.56^a^	101.2±5.34^a^	112.73±5.88v

Values within rows^(a,b,c)^ and columns^(x,y)^ with different superscripts are statistically different (p<0.05)

The mean values of ALT increased significantly (p≤0.05) at day 0 in both groups with respect to prepartum period (Table-3). Thereafter, the level of plasma ALT decreased significantly (p≤0.05) at 15 days and 30 days postpartum. Regarding AST concentration in plasma, the values remained static from −30 days to 0 day, and then, a significant decline (p≤0.05) was observed in postpartum period as compared to prepartum in both groups. There was no variation in AST and ALT values between single- and twin-bearing goats in the present study.

**Table-3:** Plasma enzymes and hormonal level in single- and twin-bearing Beetal goats during transition period.

Parameters	Group	−30 days	−15 days	Day 0	Day 15	Day 30
Alanine aminotransferase (IU/L)	Single	80.68±3.77^b^	79.24±4.55^b^	108.05±4.77^a^	90.53±4.62^c^	78.33±4.9^bd^
	Twin	73.85±3.31^b^	83.28±3.72^b^	107.79±7.15^a^	91.23±8.87^c^	69.73±5.6^bd^
Aspartate aminotransferase (IU/L)	Single	21.59±1.42^a^	20.3±0.96^a^	19.49±1.47^a^	13.77±0.91^b^	12.64±0.99^b^
	Twin	19.25±1.98^a^	20.59±1.23^a^	19.02±0.84^a^	12.87±0.7^b^	12.68±1.04^b^
Progesterone (nmole/L)	Single	36.46±3.59^a^	14.69±1.66^b^	8.58±0.95^c^	9.08±1.06^cx^	8.65±1.22^cx^
	Twin	41.11±6.40^a^	10.42±2.09^b^	6.57±1.34^b^	4.78±0.89^by^	2.79±0.57^by^
Estradiol (nmole/L)	Single	29.9±5.3^a^	27.93±7.59^a^	30.5±4.6^a^	22.32±2.1^b^	30.17±7.3^a^
	Twin	33.3±8.9^a^	23.14±10.1^b^	38.03±7.1^a^	30.97±10.1^a^	43.72±11^a^

Values within rows^(a,b,c)^ and columns^(x,y)^ with different superscripts are statistically different (p<0.05)

In the present study, the level of estrogen was higher in twin-bearing goats than in the single-bearing group from day of parturition. Between groups, the level of estrogen varies non-significantly across the days of the study, except on the day of parturition where it was significantly higher (p≤0.05) in Group 2 as compared to Group 1, indicating more stress of parturition in Group 2. A significant (p≤0.05) decrease in estrogen level was observed at −15 days; thereafter, it increased at day 0 and remained stable during postpartum period in Group 2.

The results of the present study indicated that the plasma concentration of progesterone decreased significantly (p≤0.05) at −15 days and parturition day thereafter remained stable during postpartum period in both groups. Non-significant variation of plasma progesterone was observed between the groups during whole period of study.

## Discussion

Metabolites in body are arguably the end product of all biological processes, providing immediate information about the phenotype including processes in physiological and pathological conditions. Hence, they are a great indicator of animal health, depicting underlying molecular and physiological mechanisms. Hence, they are very efficient in the diagnosis of health and diseases [8]. These molecules are of various size and nature, but among them most commonly used biochemical for diagnosis of health and disease conditions are proteins, glucose, lipids, and other associated metabolites.

An increase in glucose concentration at parturition may be due to the high concentration of glucocorticoid hormones such as cortisol, which promotes an increase in hepatic glycogenolysis and gluconeogenesis from glucose precursors as it is major energy substrate for the developing fetus, uterus, and placenta [9,10]. In the last trimester of gestation, glucose level was higher in goats carrying twins due to higher demand by fetus, as it was found a positive correlation between fetal abdominal circumference and maternal blood glucose levels [11]. Increased concentration of triglycerides in Group 2 during last fortnight of prepartum may be due to the preparation of mother for delivery. The changes in lipid metabolism have been observed in the last trimester to fulfill maternal metabolic needs, particularly energy requirement, as the glucose is utilized for fetal development [12]. The decrease in the concentration of triglycerides in the postpartum period reflects the increase in milk production, increased supply of circulating triglycerides to the mammary gland to meet the milk fat synthesis lowering the availability of free fatty acids. Lower concentrations of triglycerides at parturition and lactation when compared to prepartum were also reported [13,14] in goats and in crossbred dairy cows [15,16].

Stability in peripartum cholesterol concentration values was also reported in goats [17] and in dairy cows [16]. On the other hand, Sadjadian *et al*. [14] and Bamerny [18] observed decreased serum cholesterol concentration in the last weeks of gestation in goats. Similar results have been reported from the third last week before parturition to the 1^st^ week postpartum in dairy cows [19]. Decrease in total cholesterol and triglyceride from 0 to 45^th^ day postpartum in Surti goats indicates that the animals utilized the lipids for the supply of energy for milk production and the high glucose level on 0 day indicates that the animals were in positive energy status [20].

Similar results of the protein concentrations were also observed at the end of gestation and at parturition and the return to normal values postpartum in dairy goats [21]. This may be due to the migration of globulins directed at colostrum synthesis [22]. An increase in albumin concentration at parturition might be due to higher albumin synthesis by liver or to a decrease of plasma volume masked by hypoglobulinemia [3]. The findings of the present study of the increase in total protein and globulin in postpartum are similar to that previously reported [2]. The significant increase in plasma urea concentration observed in the prepartum days in both groups was probably due to the increase in protein catabolism to meet energy requirement and deamination of amino acids [23]. The decrease in plasma urea concentration around parturition may be associated with the decline in feed intake due to stress and hormonal changes during the kidding. However, a significant increase in blood urea nitrogen during the postpartum period has been reported in different goat breeds [14].

AST activity is an indicator of liver function in periparturient animals, having similar AST variation in Saanen goats [6]. High energy demand from newborn during lactation leads to increased gluconeogen­esis, which may be responsible for causing an increase in the serum activity of this enzyme, as a consequence of the increase in hepatic metabolism [24]. Similar results have been reported by Sadjadian *et al*. [14] in goats during lactation in relation to prepartum.

The decrease in sodium in the last week of gestation and potassium on the day of parturition and 1^st^ week postpartum was also observed by others workers in goats [18]. Waziri *et al*. [25] also did not observe alterations of these variables in dairy goats. Drainage of electrolytes during partum in fluid or loss through colostrum may lead to their alteration in blood plasma.

Higher level (overall 11.59% or higher) of estrogen was observed in twin pregnancy in Surti goats as compared to single-bearing goat on all test days although it was not significant [26]. This increase is also physiological, as estrogen is required for uterine contractions during parturition. Various physiological, endocrine, and biochemical changes occur in goats during transitional phase to cope up the nutritional requirement during lactogenesis period [27]. Prepartum surge in estrogen on day of kidding is essential for starting parturition, i.e., uterine contraction and providing sympathetic stimulus for oxytocin and triggering prostaglandin release for myometrial contraction. However, low level of estrogen was reported in twin pregnancy as compared to singlet pregnancy in crossbred goats [28]. The decline in progesterone concentration is physiological due to the destruction of the corpus luteum of pregnancy and the decreased level of progesterone released from the placenta [28,29]. Changes in analytes during pre- and post-parturition stages are not necessarily indicative of disease but may just reflect physiological variations.

## Conclusion

Changes in some biochemical parameters were significant during the transition period in both twin- and single-bearing goats but are within the normal physiological limits defined for the species. Most important changes are in relation to energy metabolism molecules, indicating that better management of body energy level during transition phase is required to get optimal production both from young ones and their mother. Understanding the effects of twinning during transition period will also be helpful to develop better managemental practices for both mother and kids.

## Authors’ Contributions

JM conceptualized and designed experiment, estimated biochemical and enzymes; SS and MKR were involved in sampling, estimation of hormones and electrolytes, statistical analysis, edited, and revised the final draft of manuscript. All authors read and approved the final manuscript.
